# Combining intracellular selection with protein-fragment complementation to derive Aβ interacting peptides

**DOI:** 10.1093/protein/gzt021

**Published:** 2013-05-24

**Authors:** Nicola Acerra, Neil M. Kad, Jody M. Mason

**Affiliations:** The School of Biological Sciences, University of Essex, Wivenhoe Park, Colchester CO4 3SQ, UK

**Keywords:** Alzheimer's disease, β-amyloid, peptide interactions, protein-fragment complementation, β-sheets

## Abstract

Aggregation of the β-amyloid (Aβ) peptide into toxic oligomers is considered the primary event in the pathogenesis of Alzheimer's disease. Previously generated peptides and mimetics designed to bind to amyloid fibrils have encountered problems in solubility, protease susceptibility and the population of small soluble toxic oligomers. We present a new method that opens the possibility of deriving new amyloid inhibitors. The intracellular protein-fragment complementation assay (PCA) approach uses a semi-rational design approach to generate peptides capable of binding to Aβ. Peptide libraries are based on Aβ regions responsible for instigating amyloidosis, with screening and selection occurring entirely inside *Escherichia coli.* Successfully selected peptides must therefore bind Aβ and recombine an essential enzyme while permitting bacterial cell survival. No assumptions are made regarding the mechanism of action for selected binders. Biophysical characterisation demonstrates that binding induces a noticeable reduction in amyloid. Therefore, this amyloid-PCA approach may offer a new pathway for the design of effective inhibitors against the formation of amyloid in general.

## Introduction

Given the strong link between β-amyloid (Aβ) deposition and Alzheimer's disease (AD), a promising therapeutic approach is the prevention of Aβ aggregation. An attractive strategy is the use of small molecules that modulate Aβ aggregation (see Arkin and Wells, 2004; Doig, 2007; Berg, 2008 and references therein) and peptide-based ‘*β-sheet breakers*’ (BSBs) (Ghanta *et al.*, 1996; Soto *et al.*, 1996, 1998; Tjernberg *et al.*, 1996; Findeis *et al.*, 1999; Poduslo *et al.*, 1999; Tjernberg *et al.*, 1999; Hughes *et al.*, 2000; Gordon *et al.*, 2001; Kokkoni *et al.*, 2006; Sciarretta *et al.*, 2006). These have focused on two core amyloidogenic ‘*recognition elements*’; residues 15–21 and 25–35 of the Aβ parent peptide (Tjernberg *et al.*, 1996; Hughes *et al.*, 2000; Kokkoni *et al.*, 2006). These regions are of particular importance since they are responsible for instigating Aβ self-association and aggregation and even form amyloid in isolation (Tjernberg *et al.*, 1996). Furthermore, solid state nuclear magnetic resonance data predict these recognition elements form a β-hairpin, providing the structural basis for amyloidogenesis in the parent peptide (Petkova *et al.*, 2002; Tycko, 2011). Therefore, many strategies have focused on modification of these regions such that they retain the ability to bind Aβ but prevent amyloid formation, for example, by introducing either a blocking group or charge to these short sequences. However, issues have been identified with solubility, protease susceptibility and population of toxic small soluble oligomers. For example, Tjernberg *et al.* (1996) demonstrated that the pentapeptide Ac-KLVFF-NH_2_ (Aβ_16–20_), despite forming fibrils itself, binds residues 25–35 of Aβ_1–42_ and prevents fibril formation. Other peptides based around regions 25–35 and 38–42 have also been identified with some encouraging results (Hughes *et al.*, 2000; Hetenyi *et al.*, 2002; Fulop *et al.*, 2004; Kokkoni *et al.*, 2006). Soto *et al.* (1996, 1998) have focused on the 15–21 core recognition element with a series of peptides based on residues Aβ_17–21_. However, other groups have shown that clearance of large amyloid fibrils can lead to the population of small cytotoxic intermediates (Lowe *et al.*, 2001). Therefore, promising molecules must be capable of preventing the population of amyloid fibres while avoiding the generation of intermediary toxic species. To date, this challenge has hampered the search for BSB peptides.

**Fig. 1.**
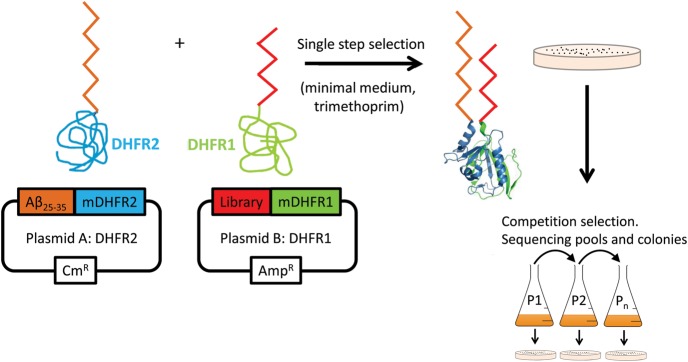
The PCA for amyloid systems. Library members that bind to Aβ_25–35_ recombine murine DHFR (PDB: 2FZJ) and lead to a colony under selective conditions (bacterial DHFR is specifically inhibited using trimethoprim). Subsequent competition selection in liquid media isolates winners of highest efficacy. Those library members that bind the target and are able to confer faster growth rates by reducing the toxic effects of the amyloid protein most effectively will be selected. Since the assay is performed in the cytoplasm of *E.coli*, any non-specific, unstable, aggregation prone (insoluble), protease susceptible members are removed.

We present a novel variation of the protein-fragment complementation assay (PCA) methodology to generate and screen peptide libraries *in vivo* (Pelletier *et al.*, 1998; Mason *et al.*, 2006; Remy *et al.*, 2007). Using the PCA approach to select peptides capable of binding to amyloid has not been published to date, although its potential use in this role has been previously speculated (Michnick *et al.*, 2011). Briefly, at the genetic level the Aβ target protein is fused to one-half of an essential enzyme, murine dihydrofolate reductase (mDHFR), with a peptide library fused to the other half. If a library member interacts with Aβ, mDHFR is recombined to yield a colony under selective conditions (Fig. 1). Since this assay is carried out via transformation of bacterial cells, the entire process is intracellular and no assumptions are made regarding the mechanism of antagonist action or which amyloid state becomes populated during selection. PCA therefore functions by screening and identifying library members that bind with the highest affinity to Aβ while additionally selecting against cells that display reduced growth rates. This reduction is due to the inherent propensity for Aβ to aggregate and the associated toxicity of fibrillar material to bacteria (Sharpe *et al.*, 2005; Soscia *et al.*, 2010). Although transformation of cells limits the realistic library size to ∼10^6^ members, PCA has other desirable attributes relative to *in vitro* library screening systems (Orner *et al.*, 2006) that aid in circumventing potential barriers to further development. For example, PCA has been previously shown to remove molecules with undesirable properties from the library, such as those that are too hydrophobic and hence insoluble, those tagged for degradation and those susceptible to protease action. In addition, non-specific off-target interactions in the crowded environment of the cytoplasm would preclude recombination of DHFR making this approach a better analogue of the *in vivo* milieu than obtainable from an *in vitro* system. There are three possible outcomes for any given library member when screened:

1. Library members bind Aβ, reduce its toxicity and recombine mDHFR, to confer cell survival.
2. Library members bind Aβ and recombine mDHFR but either populate or do not prevent population of a toxic species. These result in reduced cell growth relative to (1), or cell death.
3. Library members with no affinity for Aβ and therefore no effect on amyloid formation will not recombine mDHFR, resulting in cell death.

In outcomes (1) or (2), the Aβ target could be monomeric or oligomeric; peptides that bind either form will lead to mDHFR reassembly. We find the PCA-derived peptides to be capable of binding β-amyloid 1–42 variant (Aβ_1–42_) and reducing amyloid load. Selected peptides show promise in reversing and breaking down preformed fibrils. These studies have successfully generated a number of lead peptide sequences as a proof of principle for this generalised technique to provide a scaffold for future drug candidates.

## Material and methods

### Single-step selection PCA

*Escherichia coli* XL-1 cells were used for construction and cloning of libraries as described previously (Mason *et al.*, 2006). Firstly, pES300d-Aβ_25–35_ target and pREP4 (Qiagen; for expression of the lac repressor protein) were co-transformed into BL-21 gold cells (Stratagene) and plated onto Luria Broth (LB) agar with the appropriate antibiotics (kanamycin (Kan) and chloramphenicol (Cm)). These cells were next made electrocompetent before transformation with pES230d library plasmid. Transformed cells were plated onto three different media; 1/20th of the cells were plated onto LB agar with three antibiotics (Kan, ampicillin (Amp) and Cm) as a positive control of transformation efficiency. A further 1/20th of the solution was plated onto M9 minimal medium agar containing 1 μg/ml trimethoprim and the same three antibiotics as a negative control. Finally, the remaining 90% of transformed cells were plated onto M9 minimal agar in the presence of the three antibiotics, 1 μg/ml trimethoprim and 1 mM isopropyl-β-D-thiogalactopyranoside (IPTG), to induce expression of the two DHFR fragment fused peptides. This single-step PCA selection typically led to ∼50–100 colonies from initial libraries of 8000 and 160 000, meaning that at least 99% of all library members are removed at this stage owing to their inability to bind Aβ or rescue cell growth. Expression of Aβ_25–35_-DHFR2 target protein in the soluble fraction was verified by western blotting (see Supplementary information).

### Competition selection PCA

To increase selection stringency, growth competition experiments were undertaken. Selected colonies were pooled from the plate and grown in M9 minimal media under selective conditions (containing Kan, Amp, Cm, trimethoprim and IPTG) and serially diluted over 5–10 passages (p1–p10). Using these sequential rounds of competition selection, subtle differences in growth rate can become amplified, increasing the stringency of selection relative to the single-step method. Competition selection therefore allows the most effective 1–2 sequences to be isolated from the 50–100 Aβ binders that are initially identified during single-step selection. At each passage glycerol stocks were prepared and sequencing results were obtained (GATC Biotech, London, UK) for DNA pools as well as individual colonies. For each passage, 50 μl of liquid culture was added to 50 ml of fresh M9 minimal media, resulting in an approximate OD_600_ of 0.01. Cells were incubated at 37°C until an OD_600_ of ∼0.4 was reached (typically 2–3 days), before moving to the next passage.

### Peptide preparation

Peptides KAT, L2P1a, L2P1b, L2P2a, L2P2b and a positive control from the literature, iAβ5 (Soto *et al.*, 1996), were obtained by Peptide Protein Research (Fareham, UK) as pure lyophilised peptides and weighed using an analytical balance. Stock solutions of 1 mM concentration were subsequently dissolved in ultrapure water. At this concentration of a 2–200-fold excess of that used in inhibition and reversal experiments, no aggregation or precipitate was observed. In addition bioinformatics tools (e.g. Waltz (Maurer-Stroh *et al.*, 2010), Amylpred (Frousios *et al.*, 2009), Pasta (Trovato *et al.*, 2007), Zyggregator (Tartaglia and Vendruscolo, 2008) and Tango (Fernandez-Escamilla *et al.*, 2004)) did not predict any of the peptides to contain amyloidogenic sequences or aggregate in isolation. Finally, dye-binding experiments demonstrate that these sequences do not bind thioflavin-T (ThT) and form random-coil like species in isolation using circular dichroism (CD) (see Supplementary information).

See Supplementary information for additional methods.

## Results

We have employed protein-fragment complementation screening using peptide libraries (Mason *et al.*, 2006; Remy *et al.*, 2007) to derive peptides capable of binding to Aβ. PCA libraries were initially screened and selected on M9 minimal agar plates. Following this initial ‘single-step’ selection, colonies underwent ‘competition selection’ where they were pooled and grown before dilution. This process was repeated multiple times as ‘passages’ to select for a unique clone (Fig. 1). In this process, 1–2 binders were found to elicit the fastest bacterial growth and dominate the bacterial pool (see below). DNA sequencing continued throughout passaging until selection arrived at one discrete peptide.

### Library 1 generation

**Fig. 2.**
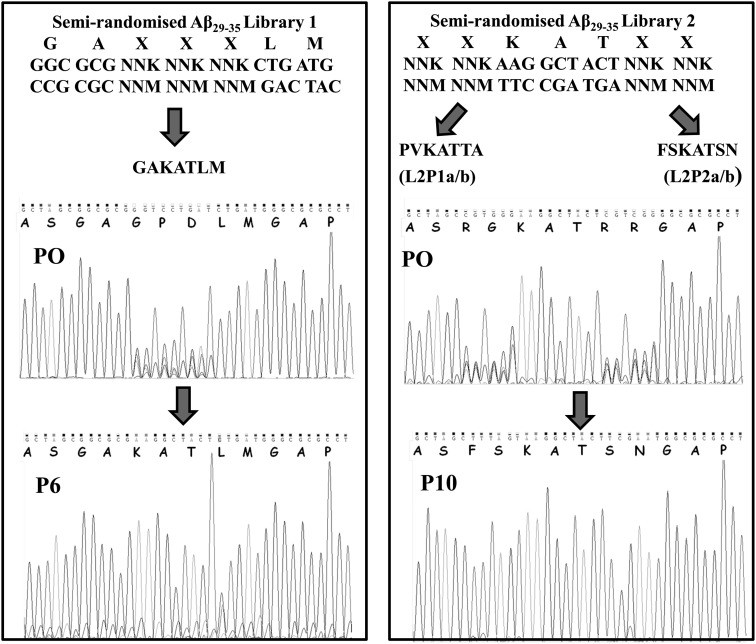
Schematic of library and sequence selection technique. The library is based on the Aβ_29–35_ region with residues 31–33 completely randomised to all amino acid options (i.e. 8000 members) in the first library. Having identified the winner GAKATLM from the first PCA, a second library with residues 29–30 and 34–35 randomised (i.e. 160 000 members) was created. This resulted in two sequences, L2P1 (FSKATSN) and L2P2 (PVKATTA). Shown also are sequencing results for single-step selections and the final passages of competition selection. For the first library, randomised residues 31–33 settled on the sequence ‘GA**KAT**LM’ as confirmed by passage 6. For the second library, two sequences were present during sequencing of individual colonies, **PV**KAT**TA** and **FS**KAT**SN**. While the second appeared to dominate the bacterial pool from passages 7–10 and was verified by sequencing individual colonies, the first was also observed in individual colonies throughout the competition selection process.

PCA was undertaken with Aβ_25–35_ target using libraries based on Aβ_29–35_ (GAIIGLM), a region of the molecule known to aggregate into toxic fibrils in isolation (Pike *et al.*, 1995; Hughes *et al.*, 2000; Hung *et al.*, 2008) and therefore a valid starting point for deriving Aβ binders capable of inhibiting aggregation. The first library incorporated three fully randomised residues at positions 31–33, generating GAxxxLM, and corresponding to a library size of 8000. Single-step selection on M9 plates was undertaken followed by competition selection in M9 liquid media, resulting in one clean sequencing result by passage six, GA_KAT_LM (Fig. 2).

### Library 2 generation

The first PCA winner (termed ‘KAT’) was used as a scaffold for the design of a second library. With residues 31–33 fixed as KAT, two residues on either sides at positions 29–30 and 34–35 were fully randomised (xxKATxx), representing a 160 000 member library (Fig. 2). Therefore, using Aβ_29–35_ as an initial design scaffold two completely unrelated sequences were subsequently derived. After 12 passages, equal preference was observed for the two sequences: L2P1 (_FS_KAT_SN_; Table I) and L2P2 (_PV_KAT_TA_; Table I). In this instance two versions of each peptide were synthesised: (i) with selected residues only (L2P1a/L2P2a), and (ii) which retained additional amino acids from restriction sites during cloning into the pES230d vector (L2P1b/L2P2b).

**Table I.** PCA-derived sequences

Name	Sequence
KAT	Ac-GA**KAT**LM-NH_2_
L2P1a	Ac-FS**KAT**SN-NH_2_
L2P1b	Ac-ASFS**KAT**SNGAP-NH_2_
L2P2a	Ac-PV**KAT**TA-NH_2_
L2P2b	Ac-ASPV**KAT**TAGAP-NH_2_

The first library consisted of 8000 members and was fully randomised with all amino acid options at positions 31–33 of the Aβ_25–35_ scaffold, resulting in the selection of ‘KAT’ at these positions after six rounds of competition selection. A second library consisting of 160 000 members was fully randomised at positions 29–30 and 34–35 with the KAT sequence from the first selection fixed. L2P1 and L2P2 sequences are based on the original KAT sequence. Addition of AS/GAP sequences reflect additional residues present in the expressed protein. Acetylation and amidation of the protein was added to mimic the polypeptide backbone and provide additional potential hydrogen-bond acceptors and donors.

### Peptide characterisation

PCA-derived peptide sequences (Table I) were synthesised and characterised using a number of experiments that included ThT dye binding, CD, oblique angle fluorescence (OAF) microscopy and transmission electron microscopy (TEM), in order to demonstrate that the peptides do not aggregate in isolation (see Supplementary information) and are able to reduce aggregation and/or breakdown preformed fibrils. In addition, growth competition assays in *E.coli* under PCA conditions in M9 media and an MTT (3-(4,5-Dimethylthiazol-2-yl)-2,5-diphenyltetrazolium bromide) assay using PC12 cells, both with the Aβ_1–42_ parent peptide, were carried out. The growth competition experiments demonstrate that peptides bind to Aβ and affect its toxicity to bacteria. MTT experiments were used to establish if the peptides also reduced Aβ toxicity in the context of mammalian cells by adding Aβ_1–42_ oligomers to PC12 cells in the presence of PCA selected peptides.

### Tht binding indicates a reduction in fibril load

**Fig. 3.**
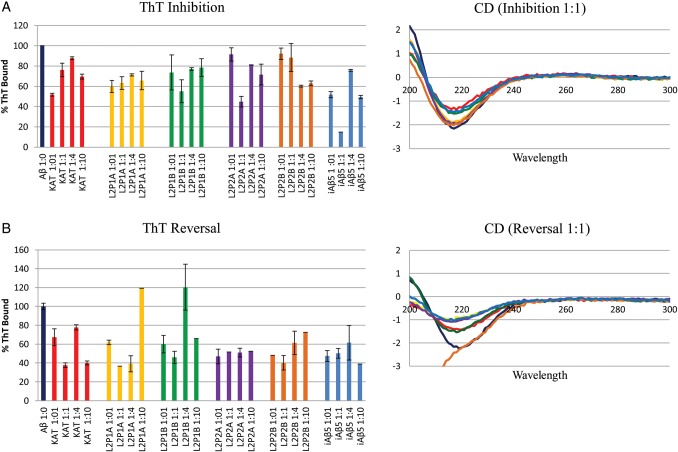
ThT inhibition and reversal data. The data (**A** and **B**) show the averaged effect of different stoichiometries of peptides KAT, L2P1a, L2P1b, L2P2a and L2P2b on the aggregation of 50 μM Aβ_1–42_.(at 3 days for the inhibition assay and at 6 days for reversal assay). Shown also are CD spectra for inhibition and reversal samples at 3 days post-mix. The assay is performed at 10 μM Aβ_1–42_ concentration.

To determine the ability of PCA-derived peptides to reduce fibril assembly (inhibition) and/or breakdown preformed fibrils (reversal), ThT binding was used to quantify amyloid species. Firstly, Aβ_1–42_ was rendered monomeric (Zagorski *et al.*, 1999) and aggregated into amyloid by resuspending and incubating at 37°C. For the inhibition assay, peptides were added on Day 0 and tested daily over a 3-day period. iAβ5, known to perform well in ThT assays, was also used as a positive control peptide (Soto *et al.*, 1998). Figure 3 shows the average of four different Aβ : peptide ratios. The ThT signal was reduced in all instances indicating that peptides are able to bind Aβ_1–42_ and reduce aggregation levels. As expected, the positive control iAβ5 peptide (Soto *et al.*, 1998) was able to reduce the ThT signal by a comparable amount. At increasingly lower sub-stoichiometric ratios, we observed progressively reduced activity consistent with a general dose dependency (see Supplementary information).

To measure the effect on the reversal of preformed fibrils, the peptides were added after 3 days of Aβ_1–42_ fibril growth. The ThT assay results showed a similar reduction in bound ThT as the inhibition assays, suggesting that peptides are able to bind Aβ_1–42_ and reverse its aggregation. The positive control peptide (iAβ5) again demonstrated a similar reduction in ThT binding, suggesting that PCA selected peptides compare favourably. Interestingly, the degree of inhibition and reversal tended to zero as the Aβ_1–42_ : peptide stoichiometry was reduced (see Supplementary information). At sub-stoichiometric ratios as the peptide dose tend towards zero, the progressively reduced activity observed again demonstrated a dose dependency (see Supplementary information).

### CD demonstrates a reduction in β-sheet content

Since amyloid fibrils are predominately β-sheet, we have used CD spectroscopy to provide an endpoint characterisation of the aggregates formed once ThT experiments were complete. The data presented on the right-hand side of Fig. 3 show spectra with a single negative peak at 218 nm, consistent with β-sheet structure. As predicted from the ThT data above the amount of β-structure decreased in inhibition experiments (stoichiometry of 1 : 1) 3 days after incubating Aβ_1–42_ with peptides KAT, L2P1b and the positive control iAβ5 sequence. In agreement with the ThT data, there is a decrease in β-signal with peptides relative to Aβ_1–42_ alone for inhibition and reversal experiments. As expected, the iAβ5 peptide also led to a reduced β-signal. Using information in this way may help to derive and combine peptides that have different antagonist properties and are therefore synergistic when administered in combination. It should be noted that the loss in CD signal intensity for Aβ incubated with PCA-derived peptides is unlikely to be attributed to increased aggregation or consequent amyloid precipitation. This is because any peptides leading to increased precipitation from amyloid-structured insoluble deposits would also generate large increases in ThT binding. In addition, these deposits would be clearly observed in both TEM and OAF imaging experiments, where solutions were thoroughly vortexed prior to loading.

### TEM shows a reduction in amyloid fibres

Imaging experiments were performed on selected samples of Aβ_1–42_ with and without peptides to assess the presence of fibrils and their morphology (Supplementary Fig. S1). Samples were derived from those used in ThT experiments as described in the materials and methods section, to allow for the direct comparison of results. Fibrils for Aβ_1–42_ were observed with a diameter of ∼10 nm (Supplementary Fig. S1). Addition of peptide caused a loss of observed fibrils, supporting the reduction in ThT binding experiments. Smaller poorly defined particles could be observed for 1 : 1 mixtures with KAT and L2P1a. No fibrils were observed for 1 : 1 mixtures with L2P1b, L2P1a or iAβ5.

### Fluorescence microscopy indicates a reduction in the quantity of large amyloid fibres

**Fig. 4.**
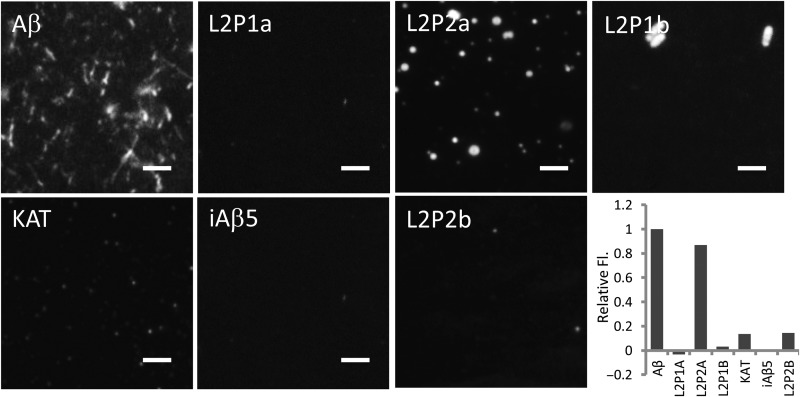
OAF microscopy. During reversal experiments, Aβ_1–42_ was grown alone for 3 days, after which peptide was added at a stoichiometry of 1 : 1 and followed by a further 3 day incubation to assay for peptide-induced reversal of amyloid deposition. Each sample was then imaged by fluorescence microscopy and panels showing representative images obtained. To quantify amyloid deposition the mean grey value over a 256 × 256 area randomly chosen for five separate images is plotted as fluorescence intensity. Each data point is scaled to overcome the ‘background noise’ by taking Aβ (1 : 0) as the maximum and iAβ5 as the minimum (i.e. (signal-iAβ5)/(Aβ-iAβ5)). This defines the range over the positive and negative controls. It can be clearly observed that all peptides except L2P2a are strongly inhibitory for this reversal assay. The scale bars represent a distance of 2 μm. OAF microscopy data for inhibition experiments are shown in Supplementary Fig. S2.

Samples used in ThT and CD experiments were also imaged using OAF microscopy for both inhibition and reversal experiments (Kad et al., 2010). To prevent bias towards any one sample, the experiment was carried out blind. This technique allows for surface associated and stacked aggregates of amyloid fibres to be directly imaged. It was therefore possible to assess the amount of protein deposited as amyloid and its morphology. For the reversal assay, Fig. 4 shows representative images of Aβ_1–42_ deposited onto the surface, as well as the effect of peptides added after 3 days incubation of Aβ_1–42_ alone. To further quantify the amount of amyloid deposited, we analysed the mean fluorescence value for each condition using ImageJ (NIH, USA) over a number of randomly chosen 256 × 256 regions and found a similar correlation. Consistent with ThT data, KAT demonstrates a major loss of fibrils present in the Aβ_1–42_ sample, but indicates a large change in morphology and that some smaller deposits do remain, with others either removing fibrils or changing their morphology.

OAF microscopy data for inhibition experiments demonstrated that fibrils are present in Aβ_1–42_, L2P1a and L2P1b samples, with others showing strongly diminished levels of ThT bound protein (Supplementary Fig. S2). Taken collectively OAF, TEM, ThT and CD data suggest that KAT is more effective at inhibiting amyloid formation, while L2P1a is more effective at reversal.

### Aβ slows bacterial cell growth

**Fig. 5.**
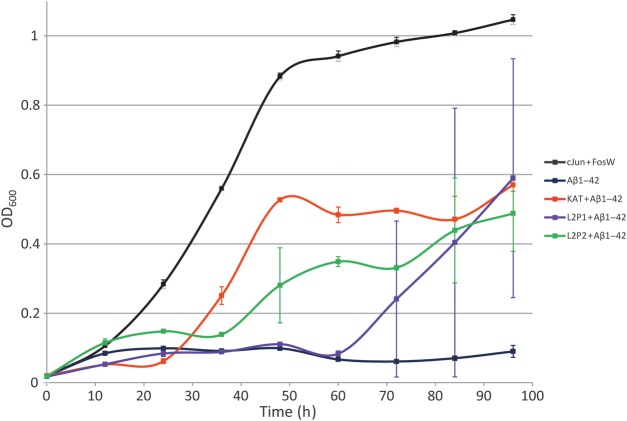
Growth competition assays with Aβ_1–42_ target. To confirm that Aβ_1–42_-DHFR2 impedes the growth rates of *E.coli*, and to ascertain that peptides are able to reverse the effect of Aβ_1–42_-DHFR2 in causing reduced bacterial growth rates, growth competition experiments were undertaken in M9 liquid media as for growth competition experiments the during PCA selection process. In these experiments cells expressed either (i) Aβ_1–42_-DHFR2 + Aβ_1–42_-DHFR1 (dark blue), (ii) a non-toxic control consisting of cJun-DHFR2 + FosW-DHFR1 (black), (iii) Aβ_1–42_-DHFR2 + peptide-DHFR1 (red, green, purple) in *E.coli.* All three peptides led to significant growth rates relative to (i) in the order KAT > L2P2 > L2P1. For growth competition assays with Aβ_35–35_ target see Supplementary Fig. S6.

The effect of peptides on the growth of *E.coli* harbouring Aβ_1–42_-DHFR2 target and peptide-DHFR1 fusion plasmids as present in the final PCA selection round was ascertained. Cells were grown from a starting OD_600_ of 0.02 under PCA conditions in M9 containing Amp, Cm, Kan, trimethoprim and IPTG for protein expression. Cell growth rates were consequently monitored over a period of 4 days as an indicator of cell health and efficacy of peptide binding (Fig. 5). Cells expressing Aβ_1–42_ + Aβ_1–42_ (dark blue line) were found to grow poorly relative to a non-toxic control (expressing cJun + FosW (Mason *et al.*, 2006)—black line). This is likely due to Aβ_1–42_ self-associating (Sharpe *et al.*, 2005) despite being soluble in western blotting experiments (see Supplementary Fig. S4), suggesting that the molecule populates a toxic and soluble oligomeric state during PCA. Therefore, this offers a method to directly assess the efficacy of an interacting peptide. The growth rate of cells expressing Aβ_1–42_ in the presence of KAT (red line), L2P1 (purple line) or L2P2 (green line) were much improved relative to Aβ_1–42_ expressed in isolation, indicating the toxicity of Aβ_1–42_ self-association is reduced by expression of the PCA selected ‘winner’ sequences. The order of improved growth was KAT > L2P2 > L2P1. After 4 days of growth all three converged on OD_600_ values of ∼0.5, much greater than ∼0.1 for cells expressing Aβ_1–42_ + Aβ_1–42_. The same experiment was repeated with Aβ_25–35_ used in the PCA library screening and selection and again demonstrated that cells expressing Aβ_25–35_ + Aβ_25–35_ grew at reduced rates relative to cells expressing Aβ_25–35_ in the presence of KAT, L2P1 or L2P2 (Supplementary Fig. S6).

## Discussion

A PCA approach has been combined with semi-rational design and peptide library screening to identify molecules capable of binding to Aβ, lowering amyloid levels and increasing bacterial growth rates. By focusing libraries around the Aβ_29–35_ sequence we have identified small peptides capable of binding to the Aβ_25–35_ region that is known to aggregate in isolation. Selection was iterative; the second library used the first PCA ‘winner’ as a design template, enabling a second round of peptides to be developed with no resemblance to the Aβ_29–35_ template. Collectively the data demonstrate that all peptides reduce amyloid levels; information from ThT and CD indicate that PCA-derived peptides are able to prevent Aβ_1–42_ fibril formation and reverse preformed fibrils. In general, the most effective molar ratio was 1 : 1, perhaps reflecting the expression system where selection would be predicted to occur at approximately equimolar levels of peptide : Aβ. In addition, as the molar ratio was progressively lowered the reduction in ThT bound was lowered as well, indicating an associated dose dependency.

A number of approaches were employed to study the effectiveness of the peptides. OAF and TEM verified the observations from ThT and CD. These imaging experiments identified much less amyloid and a distinct change in the fibril morphology, indicating that peptides cause a significant reduction in size of oligomeric species produced. Since ThT binds to many different oligomeric forms of Aβ and CD measures only an averaged global signal of β-sheet content, direct imaging offers the best indication of an effect upon Aβ. OAF microscopy experiments suggest that KAT and L2P2b are capable of substantially preventing amyloid formation, but that all peptides result in reduced size assemblies, demonstrating that to varying degrees all are able to reverse formation of preformed fibrils. Bacterial growth experiments under PCA conditions showed increased growth rates relative to cells expressing the self-associating Aβ_1–42_ or Aβ_25–35_ in isolation, indicating that binding of peptides to their Aβ targets reduces the overall cellular toxicity. Therefore, overall the data support the hypothesis that amyloid deposits are being broken down into smaller species and that peptides derived in this study compare favourably with the previously reported iAβ5 inhibitor (Soto *et al.*, 1998). The fact that some ThT fluorescence remains, along with β-sheet signal in the CD and some deposits are via imaging, suggests that peptides bind to oligomeric rather than monomeric Aβ, which would not be predicted to represent a highly structured binding epitope. In the MTT assay, no peptide improved cell viability to any significant amount (Supplementary Fig. S8). However, it should be noted that since PCA is undertaken inside bacteria it is possible that intracellular selection of peptides that are non-toxic to *E.coli* may not be as effective when transferred into a mammalian cell system. There, exposure to different off-target interactions and a different protease pool will occur. In addition it is necessary for the peptide to function in the extracellular space, where Aβ is known to aggregate in the brain. This may explain the discrepancy between growth rates in *E.coli* and efficacy in MTT experiments on peptides incubated with PC12 cells. Nonetheless these data clearly show that amyloid-PCA is capable of deriving peptides that can interfere with amyloid fibrilisation; further refinement of peptide properties could allow for the breakdown of these species into benign fragments.

With the first library winner used as a design template for the second library, it was hoped that an improvement in inhibition properties would be seen; however, all of the biophysical and biochemical data suggest no further improvement. Nonetheless, it may be possible that the future derivation of first generation peptides could show improvements in such second generation libraries. Also, further modifications employing iterations of ‘Truncation, Randomisation and selection’ (TRaSe) (Crooks *et al.*, 2011) may assist in arriving at molecules of reduced size, but comparable efficacy while fulfilling some of the traditional requirements for small molecules (Lipinski *et al.*, 2001; Lipinski, 2003; Mason, 2010). Finally, although we have not directly quantified the affinity of binding, drugs with low binding affinities (i.e. μM rather than nM–pM) may still hold considerable therapeutic promise, since aggregation is a kinetic process and therefore binding to correctly folded molecules or species present during the initial nucleation step will considerably slow amyloid conversion rates. Thus in patients newly diagnosed with AD, administering drugs that target small soluble oligomers may aid in slowing the conversion to insoluble plaques; for example, antibodies that target Aβ dimers have be shown to be effective in preventing inhibition of long-term potentiation (Klyubin *et al.*, 2008).

In this study, we have developed a straightforward high throughput *intracellular* bacterial selection approach to derive a range of peptides that bind Aβ_1–42_. By using a number of techniques, we demonstrate the differential capacity of each peptide to prevent and/or reverse fibril formation. Although at present the effect of these peptides is modest, they provide a starting point for further refinement in more complex mammalian systems. Using this approach it is envisaged that peptide inhibitors can be developed with reduced protease susceptibility and smaller size. Both may be achieved via the introduction of non-natural amino acids or main-chains, or via introduction of non-peptidic restraints that mimic or reinforce the natural secondary structure (Mason, 2010; Rao *et al.*, 2013). Other desirable properties such as improved blood–brain barrier penetration might be achieved via fusion to cell penetrating peptides (Morris *et al.*, 2008) or by decreasing the molecular weight (Lipinski *et al.*, 2001). All of these strategies may offer promise in the search for molecules targeted at slowing or even preventing the onset of AD.

## Supplementary data

Supplementary data are available at *PEDS* online.

## Funding

J.M.M. thanks Alzheimer's Research UK for a pilot project grant (ART/PPG2008B/2) and AgeUK for a New Investigator Award (#304). J.M.M. is the recipient of a Cancer Research UK Career Establishment Award (C29788/A11738) and a Wellcome Trust Project Grant (WT090184MA). N.M.K. is the recipient of a BBSRC NI award (BB/I003460/1). N.A. is funded by a University of Essex Departmental Studentship. J.M.M. and N.M.K. also thank Parkinson's UK for awarding a PhD Studentship (H-1001). Funding to pay the Open Access publication charges for this article was provided by The Wellcome Trust.

## Supplementary Material

Supplementary Data
