# Supplementary material for: Combining intracellular selection with protein-fragment complementation to derive Aβ interacting peptides
Source: Protein Eng Des Sel. 2013 May 24;26(7):463–70. doi: 10.1093/protein/gzt021 (PMC3690830; doi:10.1093/protein/gzt021)
Supplement: Supplementary Data [file supp_26_7_463__index.html]

Combining intracellular selection with protein-fragment complementation to derive Aβ interacting peptides — Supplementary Data 

# Combining intracellular selection with protein-fragment complementation to derive Aβ interacting peptides

## Supplementary Data

Supplementary Data

**Files in this Data Supplement:**

- Supplementary Data - Pdf file
