# Supplementary material for: Combining intracellular selection with protein-fragment complementation to derive Aβ interacting peptides
Source: Protein Eng Des Sel. 2013 May 24;26(7):463–70. doi: 10.1093/protein/gzt021 (PMC3690830; doi:10.1093/protein/gzt021)

Supporting Information:

PCA and expression vector cloning - PCA has been extensively used to derive protein-protein interaction antagonists of activator protein-1 (Mason *et al.*, 2009, Mason *et al.*, 2007, Mason *et al.*, 2006, Pelletier *et al.*, 1998). Briefly, mDHFR is split and one half fused to an $A\beta_{25\text{-}35}$ target peptide, and the other to the library. Target or library was fused to their respective DHFR fragment via an 11 residue glycine/serine linker (SGSSGTSSGTS) to permit flexibility and prevent steric interference. Only target binding library members bring two halves of mDHFR into close proximity, render mDHFR active and generate colony formation on M9 selective plates (Fig. 1). M9 plates contain trimethoprim to selectively inhibit bacterial DHFR. The $A\beta_{25\text{-}35}$ gene was synthesized using overlap extension PCR and cloned into the pES300d-DHFR2 vector system using NheI and AscI restriction sites. This resulted in a 6xHis-$A\beta_{25\text{-}35}$- G/S linker-DHFR2 fusion protein.

PCA Library construction - Library construction and cloning has been described previously (Pelletier, Campbell-Valois and Michnick, 1998). Briefly, in the first library, positions 31-33 of $A\beta_{29\text{-}35}$ were completely randomized using degenerate oligonucleotides containing NNK codons to create an 8000 member library. NNK was used to encode all twenty residues while removing two of three stop codons (Virnekas *et al.*, 1994). The second library was designed using the first PCA winner (‘KAT’) as a design scaffold. In this case residues 29-30 and 34-35 of KAT were randomized, again using the codon NNK to generate a library of 160,000 members (Fig. 2). The authors have used this approach extensively for the coiled coil system (Mason, Hagemann and Arndt, 2009, Mason, Schmitz, Muller and Arndt, 2006) and from all-residue randomisations have consistently observed the selection of sequences that are logical within the periodicity of the heptad repeat (i.e. HPPHPPP, where H and P are hydrophobic and polar residues respectively), suggesting that there are no specificity issues associated with this assay. Indeed, very few false positives have been observed using the approach. Negative controls are regularly undertaken on plates or liquid media that lack IPTG, indicating that overexpression of the two fusion proteins driven by the lac operon is absolutely required to provide DHFR activity.

Aβ Peptide preparation - $A\beta_{1\text{-}42}$ was purchased as a pure recombinant peptide from rPeptide (Stratech) and was used for all of the experiments described. Prior to use, the peptide was treated to three rounds of dissolution in hexofluoro-2-propanol (HFIP), sonication, drying, dissolution in trifluoroacetic acid (TFA), sonication and drying, according to the Zagorski protocol (Zagorski *et al.*, 1999), and then aliquoted into appropriately sized batches for subsequent assays and dried via lyophilisation before being dissolved in 10mM potassium phosphate buffer (pH 7.4) to generate a final concentration of 50 μM. TFA/HFIP treatment is used to ensure that amyloid growth always proceeds from the same monomeric state, thus reducing errors in amyloid formation measurements.

Western Blot Analysis– To demonstrate that either A$\beta_{25\text{-}35}$-DHFR2 of A$\beta_{1\text{-}42}$-DHFR2 fusion peptide was expressed as a soluble protein during PCA selection a western blot experiment was undertaken. In this experiment single colonies were picked into 5 ml 2xYT medium with the appropriate antibiotic and incubated at 37°C. Cells were induced with 1 mM IPTG at OD_{600} = 0.7 and incubated at 37°C overnight. The cells were next lysed using lysozyme and sonicated. The cells were centrifuged at 13000 rpm for 5 minutes to separate soluble and insoluble fractions. Each fraction was mixed with 6x loading buffer and 10 µl of each was loaded onto a 15 % SDS-PAGE gel. The gel was run for 1 h and 30 minutes at 150V before being blotted onto an Immobilon-P Transfer Membrane (Millipore) and probed with anti-His polyclonal rabbit antibody (Cell Signalling Ltd) directed at the N-terminal 6xHistag on the Aβ-DHFR2 fusion protein using a 1/1000 dilution. Reactive protein bands were visualized with anti-rabbit HRP conjugate antibody (AB-CAM Ltd) at a dilution of 1/10000. The A$\beta_{25\text{-}35}$-DHFR2 and A$\beta_{1\text{-}42}$-DHFR2 fragments migrated at the expected sizes of ~12.5 kDa and ~13.5 kDa respectively (Figure S4).

Thioflavin T Assays - ThT inhibition assays were performed with 50 µM Zagorski treated (Zagorski, Yang, Shao, Ma, Zeng and Hong, 1999) monomeric A$\beta_{1\text{-}42}$ in 200 µl of 10 mM potassium phosphate buffer, pH 7.4, with or without each peptide at a concentration of 5 µM (for 1:0.1 molar ratio), 50 µM (for 1:1 molar ratio), 200 µM (for 1:4 molar ratio), and 0.5 mM (for 1:10 molar ratio). In addition, for sub-stoichiometric experiments (Suppl. Figure 7) 0.5 µM (1:0.01 molar ratio), 50 nM (1:0.001 molar ratio) and 5 nM (1:0.0001 molar ratio) were also included to demonstrate progressively reduced activity as the peptide dose is increasingly lowered, thus demonstrating a trend of dose dependency. During ThT experiments sufficient target peptide was lyophilized, dissolved (Selkoe, 2002), and thoroughly vortexed as one single batch (for immediate use in all target:peptide mixes) to a concentration of 100 µM potassium phosphate buffer. All of the peptide solutions were then thoroughly vortexed to ensure complete dissolution. Finally, a 100 µl aliquot of the target solution was added to each 100 µl of peptide to give a total assay volume of 200 µl containing 50 µM target and either 5 µM, 50 µM, 200 µM and 500 µM peptide in potassium phosphate buffer. The assay mixture was vortexed and stored at 37°C for three days to induce aggregation. The ThT assay solution was prepared from stock containing 500 µM ThT. The stock was aliquoted and kept frozen until required. It was then allowed to thaw at room temperature for 10 min before 25x dilution into the appropriate Tris buffer, giving the required freshly prepared ThT assay solution containing 20 µM ThT in 10 mM Tris and buffer at pH 7.4. A total of 2960 µl of the ThT assay solution was then added into 40 µl of each inhibition/reversal assay mixture, thoroughly vortexed and transferred into a fluorescence cuvette. The fluorescence of amyloid-bound ThT was measured by fluorescence spectroscopy using a Cary Eclipse fluorescence spectrophotometer; bound ThT exhibits a new excitation maxima at 450 nm and an enhanced emission maxima at 482 nm (LeVine, 1993). For the inhibition assays, the target:peptide mixtures were incubated together on day zero at 37° C. Single

ThT readings were taken on days one, two and three days, at which maximal ThT binding was found. For the reversal assays, 200 µl of 50 µM target was incubated alone at 37° C for three days before adding to the required amount of lyophilized peptide. The vortexed target:peptide solutions were then incubated at 37°C for a further three days, during which time single ThT readings were taken on post-mix day three.

Interestingly, the degree of inhibition and reversal was extremely sensitive to $A\beta_{1\text{-}42}$:peptide stoichiometry (see Suppl. Figure 7). For inhibition experiments, efficacy varied from ~10-50% with the greatest average reduction at 1:1. In addition, as expected, the positive control iAβ5 peptide (Soto *et al.*, 1998) was able to reduce the ThT signal by a comparable amount between ~50-80%. Consistent with previous studies, changes in $A\beta_{1\text{-}42}$:peptide stoichiometry altered binding in a non-systematic fashion (21). For example in reversal experiments, L2P1b exhibited ~50% reduction in bound ThT at a molar ratio of 1:1, however at 1:4 the ThT signal was increased by 20% above Aβ alone. KAT also showed a decrease in reversal at 1:1 relative to 1:4 however even at this higher stoichiometry KAT still showed ~20% reversal, and at 1:10 a reduction of ~60% similar to 1:1 was observed. The importance of examining the efficacy across a range of stoichiometries is highlighted by these results, and may also suggest an important functional role of controlling dosages. Overall, ThT results indicate that the PCA derived peptides have an inhibitory effect on amyloid assembly, but an even more pronounced effect on amyloid disassembly. No single peptide performed was ranked highest in both reversal and inhibition.

Circular Dichroism (CD) - Far-UV circular dichroism (CD) spectra were recorded on an Applied Photophysics Chrirascan at 20°C. Spectra were recorded over the 200-300 nm range at a scan rate of 10 nm/min with step size of 1 nm. Spectra were recorded as the average of two scans. Peptide (10 µM in 10 mM Potassium Phosphate buffer pH 7.4) was added to a 0.1 cm cuvette. Spectra were recorded as raw ellipticity.

Transmission Electron Microscopy - The same samples that were used in ThT fluorescence experiments were also used for preparing samples for the EM assay. In this experiment a total of 10µl of the 50µM $A\beta_{1\text{-}42}$ peptide solutions were placed onto a clean strip of parafilm alongside 10µl drops of 2% w/v Phosophtungstic Acid (PTA), pH 7 (using NaOH). Carbon-coated copper grids (400 mesh/inch; Pyser-SGI Ltd) were used either as supplied or were glow-discharged for 30s to render them hydrophilic. The EM grids were then inverted on the peptide solution drops for 30 s using inverted tweezers, carefully blotted by touching the grid edge to the filter paper, and negatively stained by inverting them on the PTA drop for 30 s. The grids were then allowed to dry overnight, after which they were ready for viewing. Transmission Electron Microscopy (TEM) was undertaken using a FEI Tecnai T12 Electron Microscope at 100 kV and a magnification of 30,000x.

TEM imaging experiments were performed on selected samples of Aβ1-42 with and without peptides to assess the presence of fibrils and their morphology (Suppl. Fig. 1). Samples were derived from those used in ThT experiments as described in the materials section, to allow for the direct comparison of results. Fibrils for Aβ1-42 were observed with a diameter of approximately 10 nm (Suppl. Fig. 1). Addition of peptide caused a loss of observed fibrils, supporting the 40-60% reduction in ThT binding experiments. Smaller poorly defined particles could be observed for 1:1 mixtures with KAT and L2P1a. No Fibrils were observed for 1:1 mixtures with L2P1b, L2P1a or iAβ5.

Oblique Angle Fluorescence Microscopy Experiments - Samples were imaged on a custom built oblique angle fluorescence system (OAF, (Kad *et al.*, 2010)). The OAF system comprised an Olympus IX50 (Vermont Optechs, VT USA) stand with a 1.45NA 100x oil immersion objective (Olympus, NH USA). The laser excitation beam at 488nm (JDSU, Photonics Solutions, UK) was expanded and then guided to a focus at the back focal plane of the objective using a custom built optical train. The beam was steered to a sub-critical angle resulting in an obliquely angled far field beam. This permits high signal to noise imaging with greater sample depth penetration. Fluorescence imaging was achieved through a 500LP dichroic and clean up filter (Chroma, VT USA) before entering an Optosplit II dual colour image splitter (Cairn, UK), and was detected using an EMCCD camera (Andor IX897, Andor, UK). The emission wavelength range used was 500-605nm, therefore both the excitation and emission wavelengths were off peak for ThT, however the image quality was excellent. All samples were pre-stained with 10μM ThT, pipetted onto a clean glass slide, air dried and then imaged in 10 mM potassium phosphate buffer, pH 7.4, supplemented with 100mM DTT to minimize photobleaching. For consistency and cross-correlation, the same samples were used for inhibition/reversal imaging as those in ThT and CD experiments. In addition, all OAF experiments were performed blind to prevent bias toward any one sample.

Growth Competition Experiments – To confirm that expression of a $A\beta_{1\text{-}42}$-DHFR2 fusion impedes the growth rate of *E. coli*, and to ascertain that peptides fused to DHFR1 are able to reverse this effect, growth competition experiments were undertaken in M9 liquid media in an identical manner to that during the PCA selection process. In these experiments cells expressed either i) an $A\beta_{1\text{-}42}$ control ii) $A\beta_{1\text{-}42}$+peptide or iii) non-toxic cJun+FosW to *E.coli* and forms a high affinity interaction, leading to significant growth rates relative to i).

3-(4,5.Dimethylthiazol-2-yl)-2,5-diphenyltetrazolium Bronide (MTT) Cell-Toxicity Assay - MTT experiments were undertaken using Rat phaeochromocytoma (PC12) cells to assess the effect of the toxicity of $A\beta_{1\text{-}42}$ and to provide a direct comparison with other studies (Kokkoni *et al.*, 2006, Shearman *et al.*, 1995, Solomon *et al.*, 1997, Wakabayashi and Matsuzaki, 2007).. PC12 cells are known to be particularly sensitive and their use in this assay is well established (Shearman *et al.*, 1994). The MTT Vybrant® MTT Cell Proliferation Assay Kit (Invitrogen) was used to measure the

conversion of the water soluble MTT dye to formazan, which is then solubilized, and the concentration determined by a colour change monitored via absorbance measurement at 570 nm. The change in absorbance can then be converted to a percentage MTT reduction which can be used as an indicator of the PC12 cell health in the assay. The assay was performed with 10µM A$\beta_{1\text{-}42}$ and varying molar ratios of peptide corresponding to 1:0.1 (0.1 µM), 1:1 (10 µM), 1:4 (40 µM), 1:10 (100 µM). PC12 cells were maintained in RPMI 1640 +2mM Glutamine medium mixed with 10% Horse Serum, 5% Foetal Bovine Serum, supplemented with a 20 mg/mL Gentamicine. Cells were transferred to a sterile 96-well plate with 30000 cells per well and experiments performed in triplicate. Briefly, different concentrations of peptides were screened in the presence of 10 µM A$\beta_{1\text{-}42}$. The required volume from peptide and target stock solutions was freeze-dried overnight. The freeze-dried peptide and A$\beta_{1\text{-}42}$ target were resuspended in 100% dimethyl sulfoxide (DMSO), each at 100x stock concentration (i.e. 1 mM, 2mM, 4 mM or 10 mM). For example for the molar ratio 1:1 a total of 5 µl from each of the resuspended peptide/DMSO and target/DMSO was mixed in a well of a 96-well preparation plate, thus giving 10 µl of 1:1 mM peptide/target concentration ratio in 100% DMSO. A total of 90 µL of RPMI media was added to the 10 µL peptide/target mixture (100:100 µM peptide/target ratio in 10% DMSO). A total of 10 µL of the 50:50 µM peptide/target mixture in 10% DMSO was then dispensed into 90 µL of media/PC12 cells, at final peptide and target concentrations of 10 µM. These were incubated for 24 h at 37 °C, 5% CO_2, prior to the addition of the MTT dye. A total of 10 µL of the day was added to each well and incubated for a further 4 h at 37 °C, 5% CO_2. A total of 100 µL of the DMSO (stop/solubilisation solution) was then added to each well and was allowed to stand for 10 minutes. The absorbance was measured at 570 nm using a 96-well Versamax tunable microplate reader.

We assessed the toxicity of extracellular A$\beta_{1\text{-}42}$ deposits on PC12 cell integrity and its amelioration by pre-incubation of A$\beta_{1\text{-}42}$ with peptides. Supplementary Figure 8 shows cell viability assays across a range of A$\beta_{1\text{-}42}$:peptide ratios relative to cells in isolation or incubated with A$\beta_{1\text{-}42}$ alone (1:0). It is clear that A$\beta_{1\text{-}42}$ is toxic to PC12 cells relative to cells incubated without the protein. However, none of the peptides improved cell viability to any significant amount, despite marginal improvements at higher molar ratios.

Structural Inferences of amyloid-peptide interactions– At the structural level, the KAT peptide represents reduced hydrophobicity and increased positive charge relative to Aβ29-35. Residues 12–24 and 30–40 of full-length Aβ have been shown to adopt β-strand conformations which form parallel β-sheets through intermolecular hydrogen bonding. The two β-sheets are brought into contact through side-chain interactions to form a steric-zipper in which side-chains between β-strands are able to interdigitate with each other (57); this is mediated by the 25–29 region that forms a β-turn. Also important are L17 and F19 which form intramolecular interactions between residues I32, L34 and V36 in the fibrillar structure and are similarly observed via 13C chemical shifts between residues

(30). Although uncertain of the exact mode of binding for KAT, we hypothesize that the positive charge and retention of hydrophobic ethylene groups in the I31K substitution may block incoming Aβ monomers on the outward growing face of the fibril, while bringing solubility to the molecule. This group may additionally prevent β-hairpin closure that is normally stabilised by a D23-K28 salt bridge (58). The G33T substitution may also assist in blocking incoming monomers on the outer face. I32A leads to a hydrophobic truncation resulting in both high propensity for sheets and helices. Along with L34, it may aid to destabilise the conformation of the outer β-sheet by blocking incoming monomers from forming I32/L34-F19 interactions (58). In this scenario the non-binding side of the β-strand Lys and Thr may generate potential electrostatic interactions and increased bulk, while on the binding side of the strand Ala disfavours the docking of additional monomers due to a reduction in hydrophobicity. Regardless of the mechanism, selection of the sequence KAT in the antagonising peptide is likely to be a trade-off between mimicking the Aβ29-35 binding region, thus permitting interaction with Aβ1-42 while also preventing recruitment of further Aβ monomers to the fibril and avoiding aggregation in isolation. For L2P1, there is a substantial increase in uncharged polar residues (A30S, L34S, M35N) with additional hydrophobic bulk at the N-terminus (G29F). L2P2 shows an increase in hydrophobicity relative to the wild type sequence (G29P, A30V, M35A), and a degree of polarity due to L34T. Along with I32A substitution of L34 for either Ser or Thr may bind Aβ1-42 and prevent β-hairpin closure by restricting F19 from contacting I32/L34 (Suppl. Fig. 3). In addition, the PCA method used here selected Pro at the N-terminus, which may hinder incoming Aβ monomers from binding to the fibril, in a mode akin to the iAβ5 peptide (25, 26). What is clear is that in all cases peptides must strike a balance between Aβ binding and peptide solubility; PCA selection ensures that these restraints are met. It should be noted that inclusion of a flexible 11 residue G/S linker on both Aβ and library permits their interaction without imposing strict geometric constraints, thereby allowing movement without steric interference between fused domains.

Supplementary Figure 1: Transmission Electron microscopy was performed on selected samples of $A\beta_{1\text{-}42}$ with and without peptide to monitor for both the presence of fibrils and their morphology. Samples were taken from the same sample used in ThT experiments to allow for the direct comparison of results. Fibrils for $A\beta_{1\text{-}42}$ were observed with a diameter of approximately 10 nm. Fibrils were clearly observed for A) Aβ in isolation. Smaller poorly defined particles were observed for 1:1 mixtures with B) KAT and C) L2P1a No Fibrils were observed for 1:1 mixtures with C) L2P1b, D) L2P1a or E) the control peptide i$A\beta$5.

Supplementary Figure 2: Oblique Angle Fluorescence (OAF) microscopy data for Inhibitor experiments undertaken at 1:1 stoichiometry of $A\beta_{1\text{-}42}$:peptide. In this experiment the same sample was used as for ThT experiments, in which $A\beta$ amyloid was grown with peptide for three days at

37°C. Each of the samples were imaged by fluorescence microscopy and panels showing representative images obtained. To quantify amyloid deposition the mean grey value over a 256x256 area randomly chosen for five separate images is plotted as fluorescence intensity. Each data point is scaled to overcome the 'background noise' by taking Aβ (1:0) as the maximum and iAβ5 as the minimum (i.e. (signal-iAβ5)/(Aβ-iAβ5)). This defines the range over the positive and negative controls. It can be clearly seen that both KAT and L2P2b are strongly inhibitory for this inhibition assay. The scale bars represent a distance of 5 μm.

Supplementary Figure 3: Solid state NMR structure of A$\beta_{1\text{-}40}$ adapted from Petkova *et al* (Petkova *et al.*, 2002). Hydrophobic residues are shown in green, polar residues in magenta, positively charged in blue, and negatively charged in red.

Supplementary Figure 4: Western blot analysis of the A$\beta_{25\text{-}35}$-DHFR2 and A$\beta_{1\text{-}42}$-DHFR2 fusion proteins. Soluble and Insoluble fractions of the bacterial cell lysate were run as an SDS-PAGE followed by immunoblotting using an Anti-His antibody. Detection of the presence of a ~12.5 kDa or ~13.5 kDa fragment in the soluble fraction corresponded to the respective fusion protein and was used to confirm that the proteins are overexpressed.

Supplementary Figure 5: Circular Dichroism spectroscopy and ThT experiments undertaken on peptides in isolation that have been incubated at 50 μM for 3 days under conditions identical to aggregation assays using A$\beta_{1\text{-}42}$. These experiments demonstrate that peptides do not bind significant amounts of ThT, and that the CD signal for all peptides is consistent with that of a random coil. They therefore indicate along with computational aggregation prediction programs (e.g. Waltz (Maurer-Stroh *et al.*, 2010), Amylpred (Frousios *et al.*, 2009), Pasta (Trovato *et al.*, 2007), Zyggregator (Tartaglia and Vendruscolo, 2008), and Tango (Fernandez-Escamilla *et al.*, 2004) that peptides do not form amyloid in isolation.

Supplementary Figure 6: Growth Competition Assays. To confirm that A$\beta_{25\text{-}35}$-DHFR2 impedes the growth rates of *E.coli*, and to ascertain that peptides are able to reverse the effect of A$\beta_{25\text{-}35}$-DHFR2 in causing reduced bacterial growth rates, growth competition experiments were undertaken in M9 liquid media as for growth competition experiments the during PCA selection process. In these experiments cells expressed either i) A$\beta_{25\text{-}35}$-DHFR2 +A$\beta_{25\text{-}35}$-DHFR1 (dark blue) ii) a non-toxic control consisting of cJun-DHFR2 +FosW-DHFR1 (black) iii) A$\beta_{25\text{-}35}$-DHFR2 +peptide-DHFR1 (red, green, light blue) in *E.coli*. All three peptides led to significant growth rates relative to i) in the order L2P2 > KAT > L2P1.

Supplementary Figure 7: ThT Inhibition and Reversal data at a range of molar ratios ranging from sub- to super-stoichiometric. A) the effect of peptides KAT, L2P1a, L2P1b, L2P2a, L2P2b and iAβ5 on the aggregation of 50μM A$\beta_{1\text{-}42}$ (at three days for the inhibition assay and at seven days for reversal

assay) at seven different stoichiometries. B) the average ThT bound for all peptides at any given molar ratio. Errors are given as the standard deviation of all errors at each molar ratio. The data show that for the three lowest molar ratios (all sub-stoichiometric; 1:0.01, 1:0.001, and 1:0.0001) the average reduction in ThT bound is minimal (86% for Inhibition and 88% for reversal). In contrast at the three highest molar ratios the reduction in ThT bound was significantly greater (67% for inhibition, 58% for reversal). The most effective average molar ratio for peptides was 1:1 which displayed ThT bound values of 57% and 44% for inhibition and reversal, approximating to 30% and 40% less than the average of the three lowest stoichiometries respectively.

Supplementary Figure 8: MTT toxicity assays using $A\beta_{1\text{-}42}$ and selected peptides using different molar ratios after 24 hours of incubation with PC12 cells. The assay was performed with 10 μM $A\beta_{1\text{-}42}$ and different concentrations of peptide, for example, 1:0.1 (1 μM), 1:1 (10 μM), 1:4 (40 μM), 1:10 (100 μM).

References

Fernandez-Escamilla A.M., Rousseau F., Schymkowitz J. and Serrano L. (2004) *Nat Biotechnol,* **22**, 1302-1306.

Frousios K.K., Iconomidou V.A., Karletidi C.M. and Hamodrakas S.J. (2009) *BMC Struct Biol,* **9**, 44.

Kad N.M., Wang H., Kennedy G.G., Warshaw D.M. and Van Houten B. (2010) *Mol Cell,* **37**, 702-713.

Kokkoni N., Stott K., Amijee H., Mason J.M. and Doig A.J. (2006) *Biochemistry,* **45**, 9906-9918.

LeVine H., 3rd (1993) *Protein Sci,* **2**, 404-410.

Mason J.M., Hagemann U.B. and Arndt K.M. (2009) *Biochemistry,* **48**, 10380-10388.

Mason J.M., Muller K.M. and Arndt K.M. (2007) *Biochemistry,* **46**, 4804-4814.

Mason J.M., Schmitz M.A., Muller K.M. and Arndt K.M. (2006) *Proc Natl Acad Sci U S A,* **103**, 8989-8994.

Maurer-Stroh S., Debulpaep M., Kuemmerer N., Lopez de la Paz M., Martins I.C., Reumers J., Morris K.L., Copland A., Serpell L., Serrano L. *et al.* (2010) *Nat Methods,* **7**, 237-242.

Pelletier J.N., Campbell-Valois F.X. and Michnick S.W. (1998) *Proc Natl Acad Sci U S A,* **95**, 12141-12146.

Petkova A.T., Ishii Y., Balbach J.J., Antzutkin O.N., Leapman R.D., Delaglio F. and Tycko R. (2002) *Proc Natl Acad Sci U S A,* **99**, 16742-16747.

Selkoe D.J. (2002) *Science,* **298**, 789-791.

Shearman M.S., Hawtin S.R. and Tailor V.J. (1995) *J Neurochem,* **65**, 218-227.

Shearman M.S., Ragan C.I. and Iversen L.L. (1994) *Proc Natl Acad Sci U S A,* **91**, 1470-1474.

Solomon B., Koppel R., Frankel D. and Hanan-Aharon E. (1997) *Proc Natl Acad Sci U S A,* **94**, 4109-4112.

Soto C., Sigurdsson E.M., Morelli L., Kumar R.A., Castano E.M. and Frangione B. (1998) *Nat Med,* **4**, 822-826.

Tartaglia G.G. and Vendruscolo M. (2008) *Chem Soc Rev,* **37**, 1395-1401.

Trovato A., Seno F. and Tosatto S.C. (2007) *Protein Eng Des Sel,* **20**, 521-523.

Virnekas B., Ge L., Pluckthun A., Schneider K.C., Wellnhofer G. and Moroney S.E. (1994) *Nucleic Acids Res,* **22**, 5600-5607.

Wakabayashi M. and Matsuzaki K. (2007) *J Mol Biol,* **371**, 924-933.

Zagorski M.G., Yang J., Shao H., Ma K., Zeng H. and Hong A. (1999) *Methods Enzymol,* **309**, 189-204.

Supplementary Figure 1

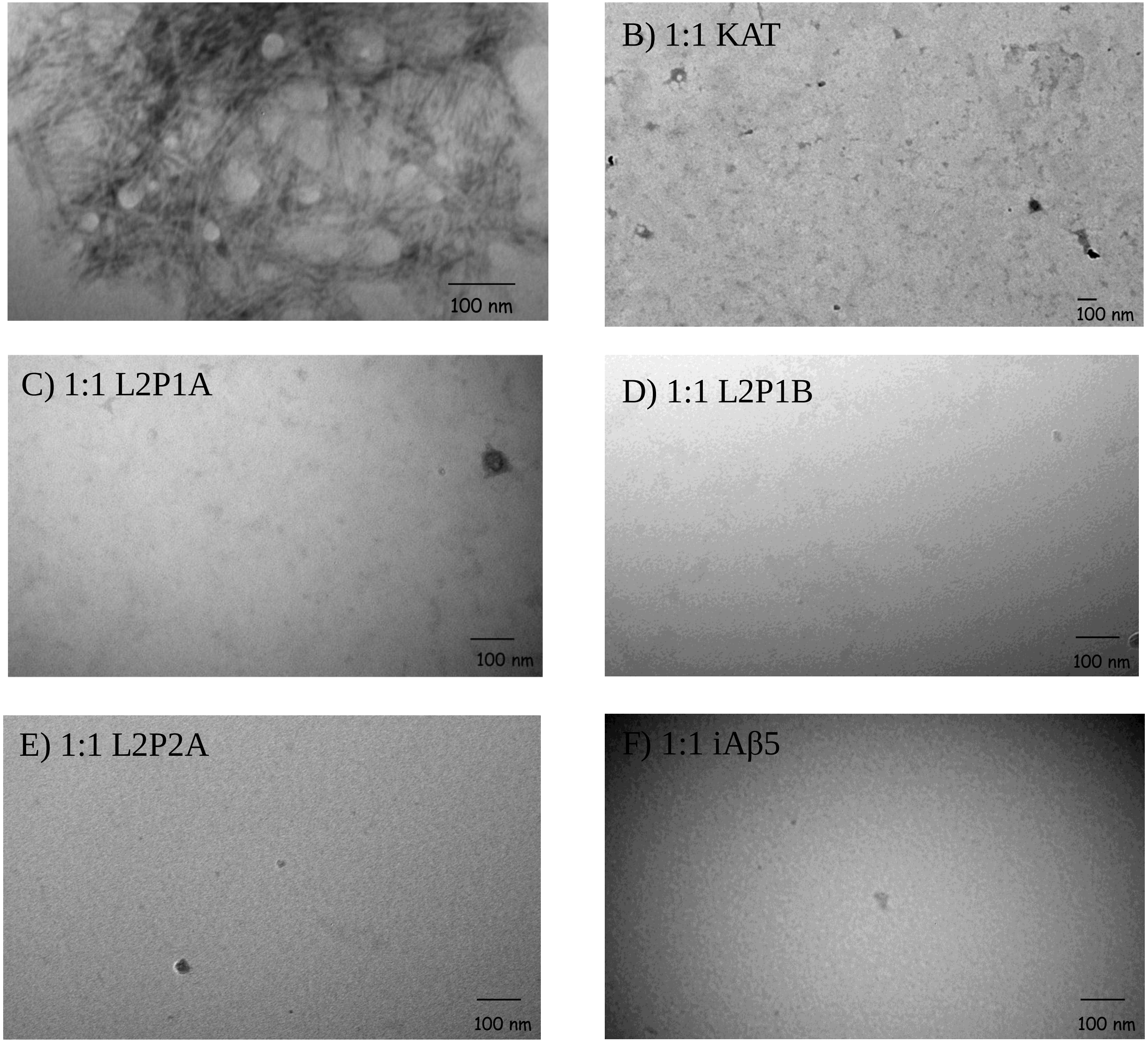

Supplementary Figure 2

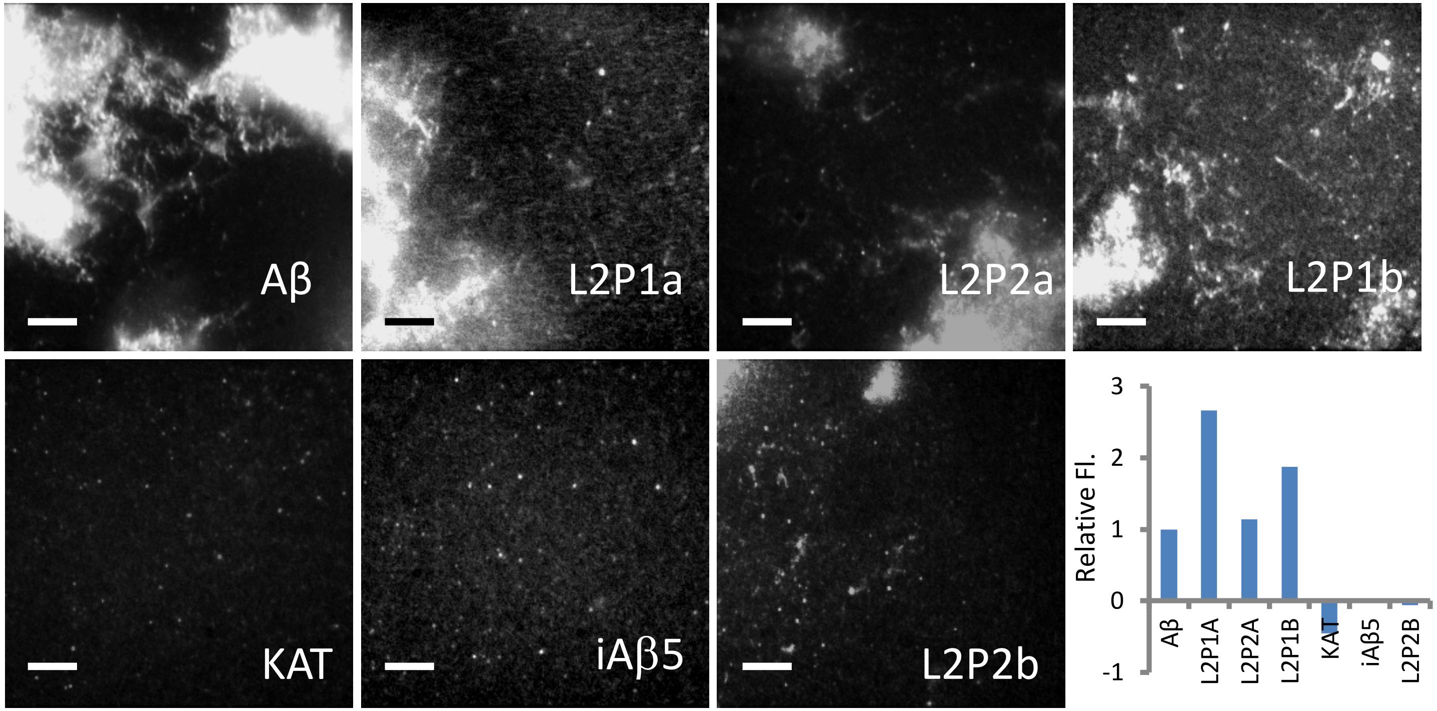

Supplementary Figure 3

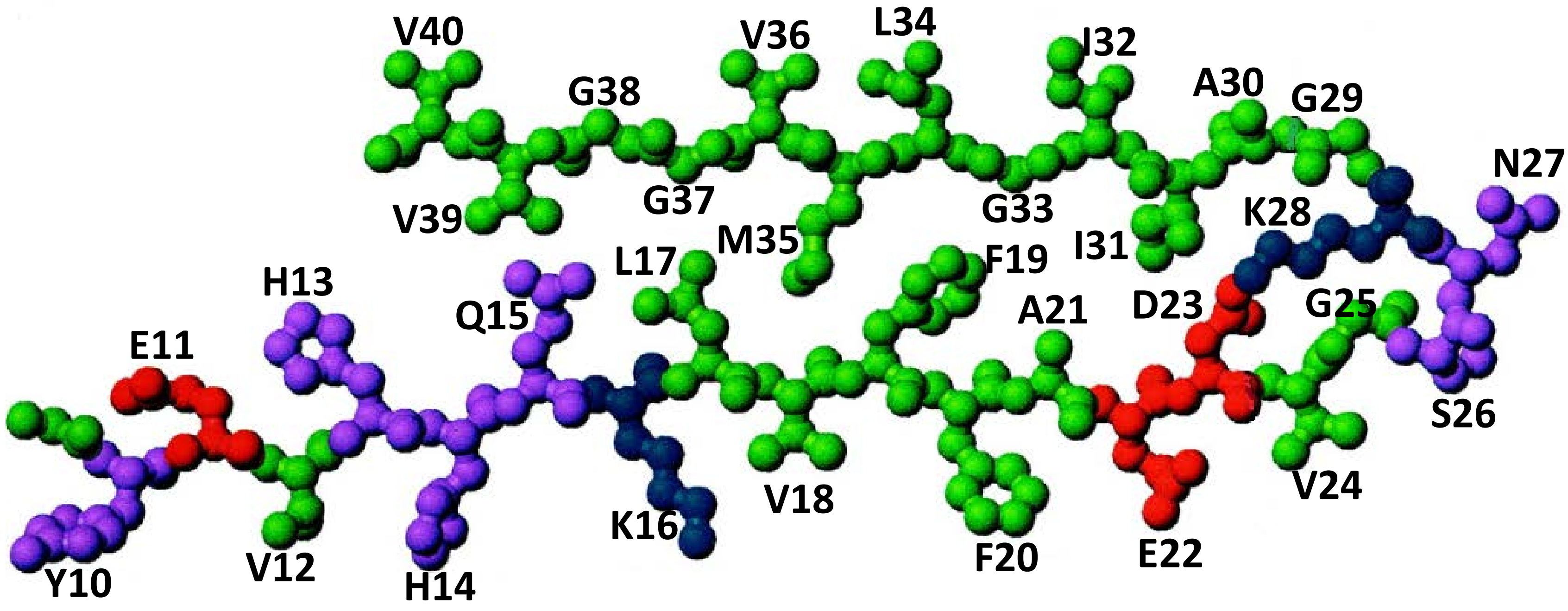

Supplementary Figure 4

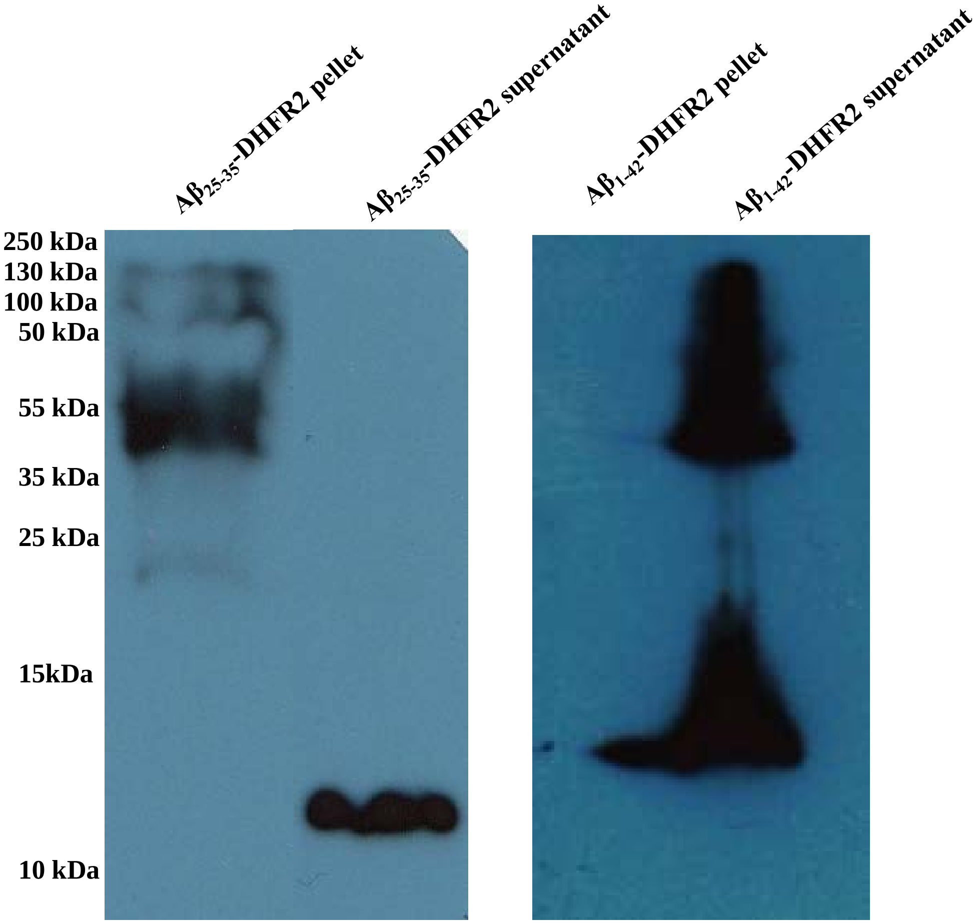

Supplementary Figure 5

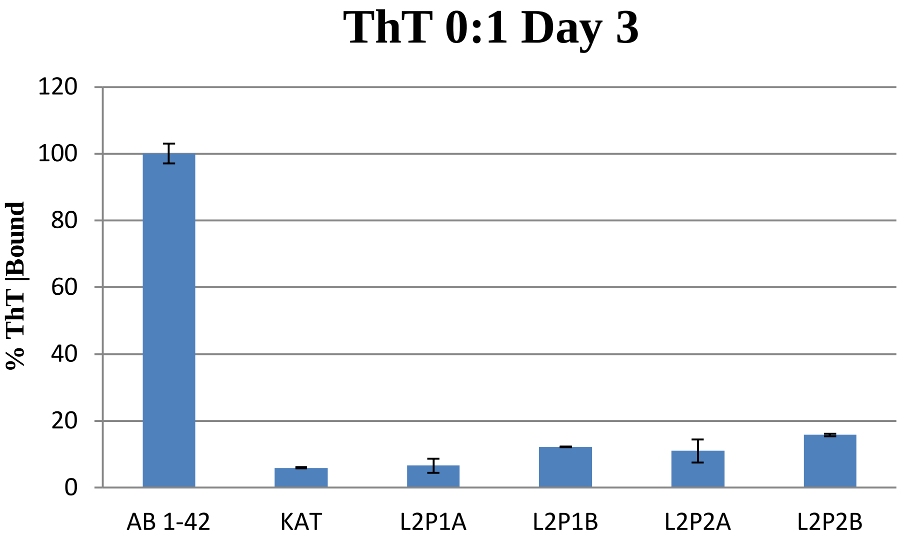

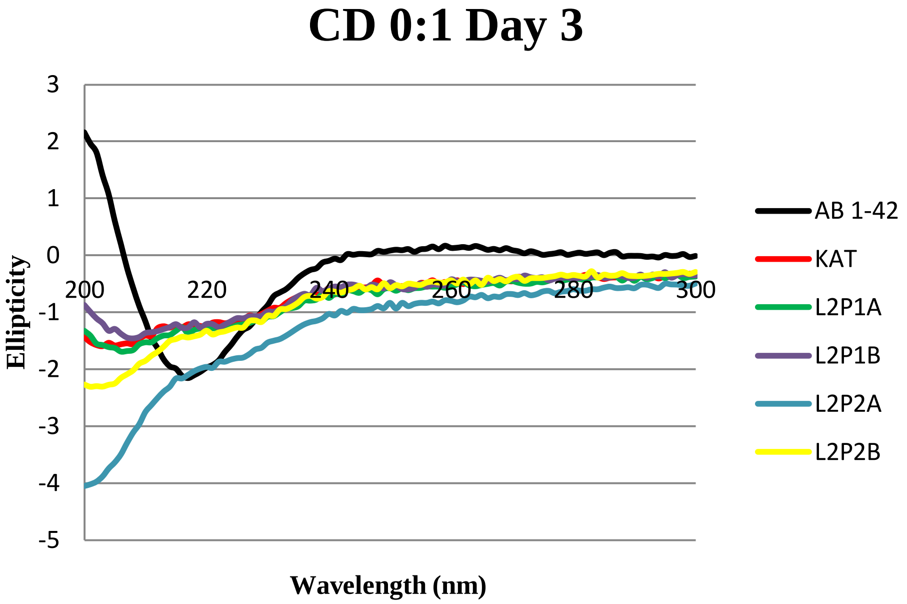

Supplementary Figure 6

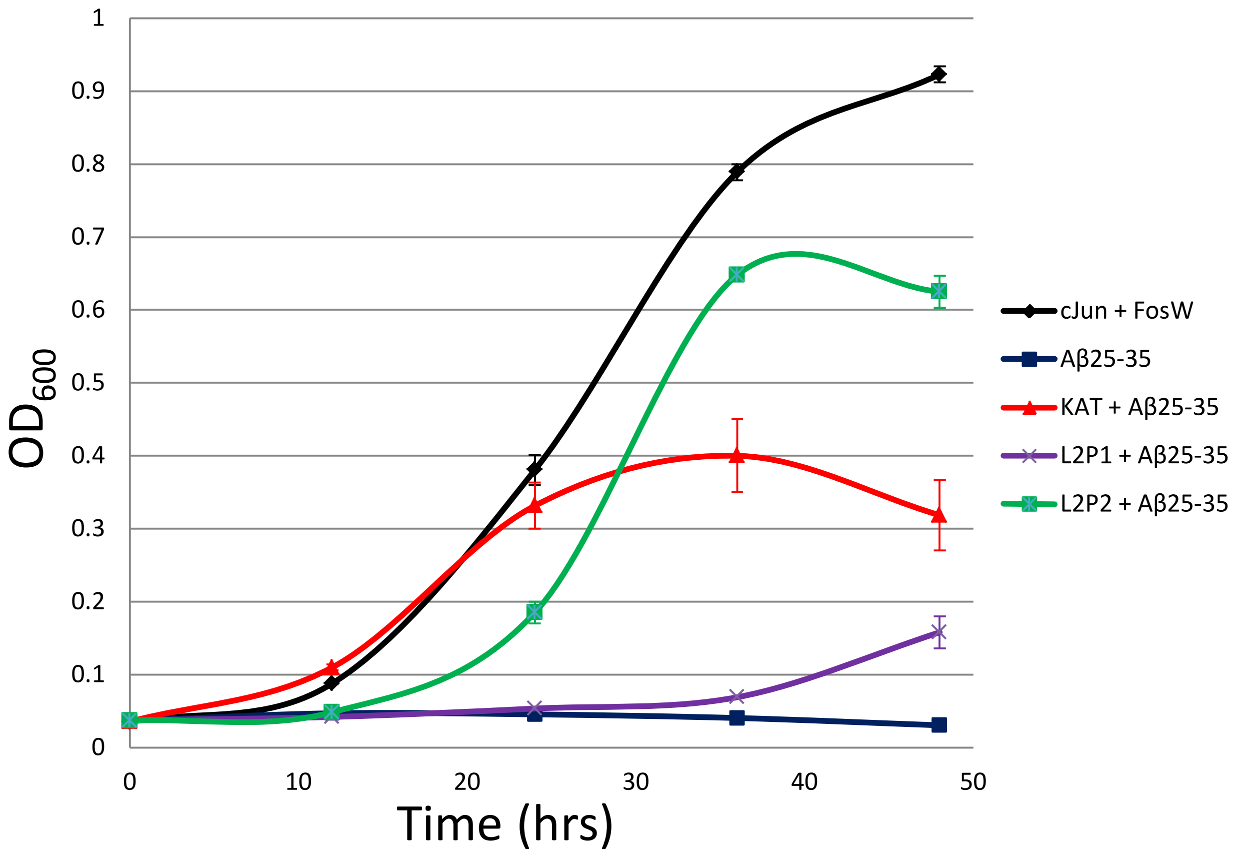

Supplementary Figure 7

A)

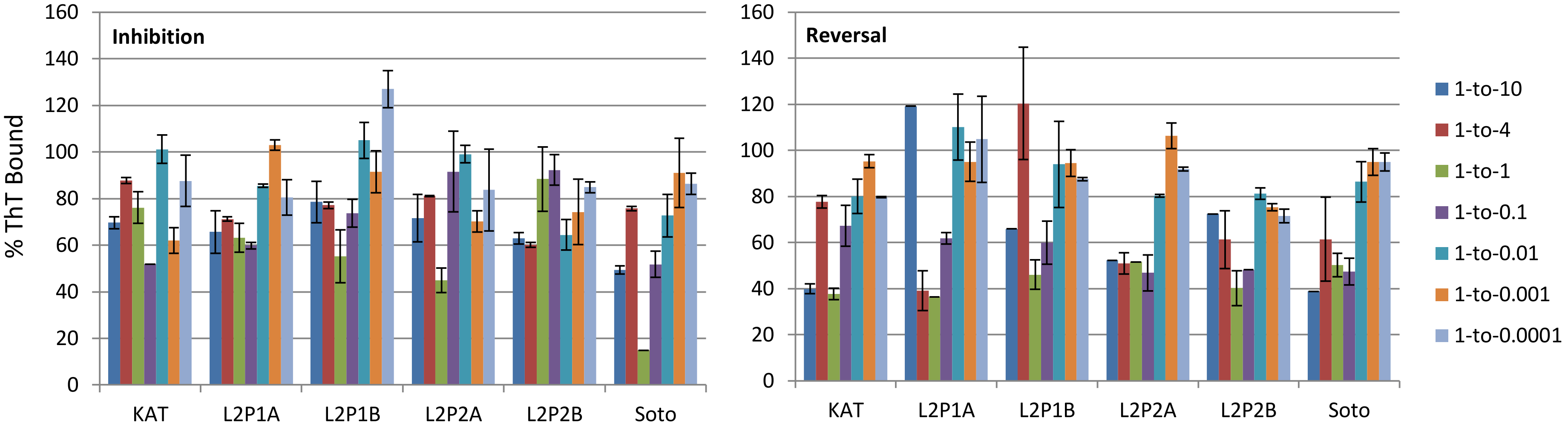

B)

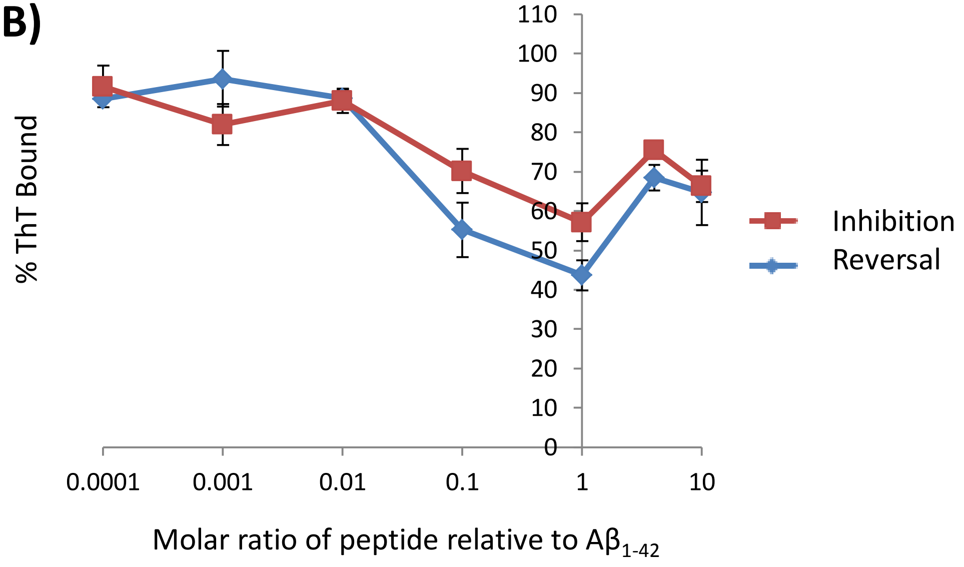

Supplementary Figure 8

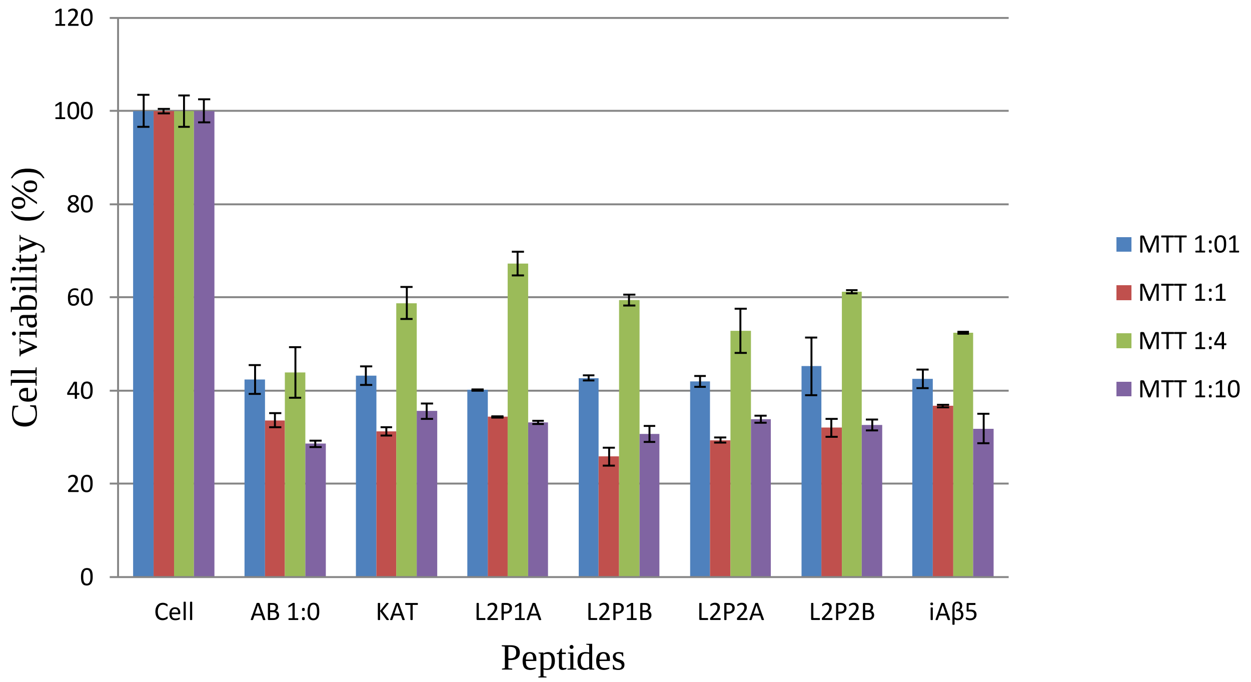

Supplement: Supplementary Data [file supp_gzt021_gzt021supp.pdf]
